# Seroprevalence of COVID-19 in Blood Donors: A Systematic Review and Meta-Analysis

**DOI:** 10.1155/2022/9342680

**Published:** 2022-07-21

**Authors:** Hassan Nourmohammadi, Ali Hasanpour Dehkordi, Amir Adibi, Seyed Mohammad Amin Hashemipour, Mohsen Abdan, Moloud Fakhri, Zahra Abdan, Diana Sarokhani

**Affiliations:** ^1^Department of Internal Medicine, Shahid Mostafa Khomeini Hospital, Ilam, Iran; ^2^Social Determinants of Health Research Center, School of Allied Medical Sciences, Shahrekord University of Medical Sciences, Shahrekord, Iran; ^3^Department of Child and Adolescent Psychiatry, Ilam University of Medical Sciences, Ilam, Iran; ^4^Young Researchers and Elites Club, Faculty of Medicine, Islamic Azad University, Yazd Branch, Yazd, Iran; ^5^Research Center for Environmental Determinants of Health, School of Public Health, Kermanshah University of Medical Sciences, Kermanshah, Iran; ^6^Traditional and Complementary Medicine Research Center, Addiction Institute, Mazandaran University of Medical Sciences, Sari, Iran; ^7^Clinical Research Development Center, Imam Reza Hospital, Kermanshah University of Medical Sciences, Kermanshah, Iran

## Abstract

**Introduction:**

Determining the prevalence of SARS-CoV-2 in blood donors makes the control of virus circulation possible in healthy people and helps implement strategies to reduce virus transmission. The purpose of the study was to examine the seroprevalence of COVID-19 in blood donors using systematic review and meta-analysis.

**Materials and Methods:**

The electronic databases PubMed, Scopus, Web of Science, and the Google Scholar search engine were searched using standard keywords up to 2022-04-26. The variance of each study was calculated according to the binomial distribution. Studies were combined according to the sample size and variance. *Q* Cochrane test and I2 index were used to examine the heterogeneity of the studies. Data analysis was performed in STATA 14 software, and the significance level of the tests was *P* < 0.05.

**Results:**

In the 28 papers examined with 227894 samples, the seroprevalence of COVID-19 in blood donors was 10% (95% CI: 9%, 11%), estimated 5% (95% CI: 4%, 7%) among men and 6% (95% CI: 4%, 7%) among women. This rate in different blood groups was as follows: A 12% (95% CI: 10%–14%), B 12% (95% CI: 10%–15%), AB 9% (95% CI: 7%–12%), and O 13% (95% CI: 11%–16%). The seroprevalence of COVID-19 in blood donors in North America 10%, Europe 7%, Asia 23%, South America 5%, and Africa was 4%; Moreover, the seroprevalence of IgG antibodies was estimated to be 23% (95% CI: 18%–29%) and IgM 29% (95% CI: 9%–49%).

**Conclusion:**

The highest prevalence of COVID-19 serum in women blood donors was among blood group O and Asia. The seroprevalence of IgG and IgM antibodies was high too.

## 1. Introduction

Severe acute respiratory syndrome coronavirus 2 (SARS-CoV-2) has led to a worldwide pandemic with millions of infected patients [1]. In March 2020, the World Health Organization (WHO) declared COVID-19 a global epidemic. COVID-19 leads to a respiratory illness that could range from severe pneumonia to mild respiratory illness. Symptoms including fever, dry cough, fatigue, headache, shortness of breath, and diarrhea are seen in these patients. However, some cases are fully asymptomatic [2]. One study found that the SARS-CoV-2 damaged organs such as the lungs, heart, kidneys, and liver, which were high in ACE2 receptors [3]. The number of cases confirmed by health care systems is of the epidemic progression indices of the disease. Nonetheless, the true burden of infection could be measured more accurately with the prevalence of the SARS-CoV-2 antibody [4, 5]. Over the past 25 years, evidence has shown that blood donors are a special class for studying subclinical states describing the prevalence and natural course of infectious diseases [6, 7]. Some studies indicate that the high rate of false-negative tests is because some factors could affect the results, such as biological sample type, insufficient collection, viral load fluctuations, and the period between blood sampling and the symptom onset [8].

Antibody detection has been considered a major point in epidemiological studies and evaluation of population control programs recently [9]. While immune responses to SARS-CoV-2 could start as soon as the first week following the symptom onset, in most infected people, seroconversion changes usually start within 10–12 days for IgM and 12–15 days for IgG. Serum IgM levels peak in two to three weeks, whereas IgG antibodies peak in three to four weeks following the symptom onset [10]. Blood donor-based zero surveillance is a powerful and cost-effective strategy bringing about valuable insights into the prevalence and infection of emerging and past infectious threats, such as West Nile virus, dengue, chikungunya, and Zika [11–16].

In a systematic review study conducted in 2021 by Milad Zandi et al., out of 12,946 patients surveyed, 7643 of them used molecular techniques, in particular, the combined RT-PCR/qPCR (qRT-PCR) technique, tested positive for COVID-19. It was confirmed in them. Among COVID-19 patients who tested positive for PCR, most showed fever or cough as the main clinical symptoms. Diarrhea, headache, and fatigue were less common among COVID-19 patients. The researchers concluded that despite the fact that the spread of the epidemic has been somewhat prevented and that it has progressed globally to vaccination and treatment, the adequacy of vaccines and treatments has not yet been determined. Therefore, early detection of infected people remains the key to limiting the epidemic [17]. Determining the prevalence of SARS-CoV-2 in blood donors enables the control of virus circulation in healthy individuals and helps implement strategies to reduce transmission, especially in the absence of seroprevalence surveys. Few studies exist on the prevalence of COVID-19 in blood donors [18, 19]. The present study aims at estimating the seroprevalence of COVID-19 in blood donors in the world.

## 2. Materials and Methods

### 2.1. Search Strategy

The study was a systematic review and meta-analysis investigating the seroprevalence of COVID-19 in blood donors throughout the world. To this end, PubMed, Scopus, and Web of Science electronic databases and the first 5 pages of the Google Scholar search engine were searched using the keywords “SARS-Cov-2, COVID-19, Coronavirus 2, Seroprevalence, Blood donors,” and their MeSH equivalents along with their compounds were searched using (AND, OR) operators with no linguistic and time constraints. The resources found were associated with 2019–2022, and the search was updated until 2022-04-26. Moreover, a reference list of all preliminary studies included in the systematic review and meta-analysis phase for the manual search was reviewed. The study used the Preferred Reporting Items for Systematic Review and Meta-analysis (PRISMA) protocol [20] for systematic review and meta-analysis. International database search strategies are listed in Table s1.

We used the population, intervention, comparator, outcomes, and setting (PICOS) strategy to carry out this systematic review and meta-analysis as follows:

  Population: the participants were healthy blood donors throughout the world with no restrictions on gender, age, blood type, or race.
  Intervention: NA.
  Comparison: NA.
  Outcome: the main outcome of the study was to estimate the seroprevalence of COVID-19 in blood donors throughout the world.

### 2.2. Eligibility Criteria

In this meta-analysis, the studies were carried out to examine the seroprevalence of COVID-19 in blood donors. To this end, the studies with nonrandom sampling, the ones reporting seroprevalence of COVID-19 in a population other than blood donors, case report studies, low-quality studies based on NOS checklist [21], nonreport of information needed for data analysis like sample number or seroprevalence of COVID-19 were excluded from the systematic review and meta-analysis process.

### 2.3. Quality Assessment

After determining the initial studies, two independent authors examined the studies qualitatively using the Newcastle Ottawa Scale Checklist. Here, a star system is used to quantitatively examine the quality of the study: for the highest quality studies, a maximum of one star is awarded for each item except for the comparison case where two stars could be assigned. According to this checklist, the papers are rated from zero (lowest quality) to ten (highest quality), and the ones with a total score of less than four are considered as low-quality studies and thus excluded. However, in the meta-analysis, we aced no studies with a score less than four [21] (Table s2). If there is disagreement among the scholars about the qualitative evaluation of studies, the third scholar eliminated the disagreement.

### 2.4. Data Extraction

The two scholars extracted data independently from the studies to minimize bias in reporting and data collection. They entered the extracted data into a checklist including the author's name, study type, age group, blood type, the total number of samples, the number of men and women, study publication year, country of study, seroprevalence of COVID-19 in total blood donors and by gender and blood type, and so on.

### 2.5. Statistical Analysis

The study used the seroprevalence of COVID-19 in blood donors to estimate the point prevalence and 95% confidence interval. The variance of each study was calculated according to the binomial distribution. Studies were combined according to the sample size and variance. *Q* Cochrane test and I^2^ index were used to examine the heterogeneity of the studies. There are three categories regarding the I^2^ index (less than 25%, low heterogeneity; 25%–75%, moderate heterogeneity; and more than 75% severe heterogeneity). The combination of heterogeneous and homogeneous studies was performed using the random effect and stable effect models in meta-analysis, respectively. The heterogeneity in our study was 99.6%, which was categorized as high heterogeneity. Therefore, in this meta-analysis, a random-effects model was used. Meta-regression was used to examine the relationship between the “seroprevalence of COVID-19 in blood donors” and the sample size OR year of publication. All statistical analyses were performed using STATA 14. The statistical level of significance was set at *P* value < 0.05.

## 3. Results

### 3.1. Study Selection Process

**Figure 1:**
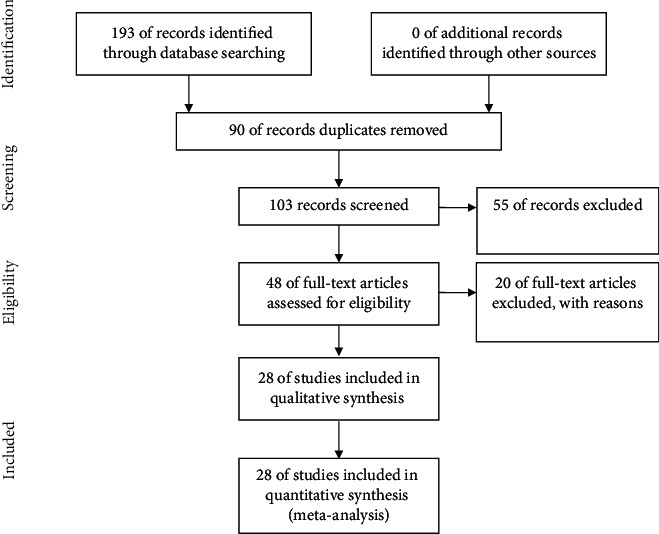
PRISMA flow diagram study.

One hundred and ninety-three papers were found by searching the above database. Moreover, 90 overlapping (repetitive) studies were excluded by reviewing the study title. The abstract section of the remaining 103 papers was reviewed and 55 were excluded based on the exclusion criteria. Out of the remaining 48 papers, another 20 papers were deleted because of the incomplete information or the lack of full text, and finally, the remaining 28 papers entered the quality evaluation stage, all of which had the quality desired (Figure 1).

**Figure 2:**
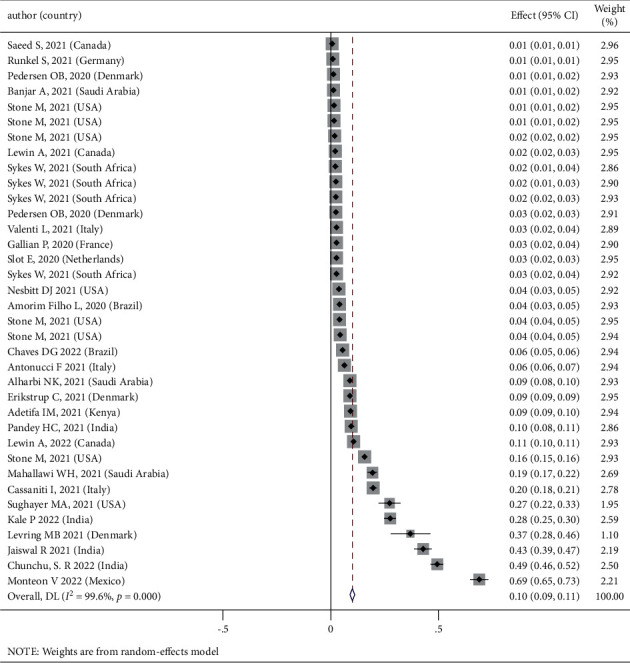
Seroprevalence of COVID-19 in blood donors and its 95% confidence interval.

The seroprevalence of COVID-19 in blood donors varied from 0.1% in the study of Ng et al. [22] to 69% in the study of Monteon et al. [23] in the 28 studies examined with 227894 samples. In this meta-analysis, the seroprevalence of COVID-19 in blood donors throughout the world was estimated at 10% (95% CI: 9%, 11%) (Figure 2). The reviewed papers' information are given in Table 1.

In the subgroup analysis, it was found that the seroprevalence of COVID-19 in blood donors was 5% (95% CI: 4%, 7%) in men and 6% (95% CI: 4%, 7%) in women. By blood group, they were reported as groups A 12% (95% CI: 10%–14%), B 12% (95% CI: 10%–15%), AB 9% (95% CI: 7%–12%), and O 13% (95% CI: 11%–16%). Furthermore, the seroprevalence of IgG antibodies was estimated at 23% (95% CI: 18%–29%) and IgM 29% (95% CI: 9%–49%).

In terms of location, the lowest seroprevalence of COVID-19 in blood donors was reported in Germany with 1% and the highest in Mexico with 69%. The seroprevalence of COVID-19 in blood donors in North America 10%, Europe 7%, Asia 23%, South America 5%, and Africa was 4% (Table 2). However, one has to note that the number of studies carried out in various countries and continents differed, and in some countries or continents only one study had been carried out.

**Figure 3:**
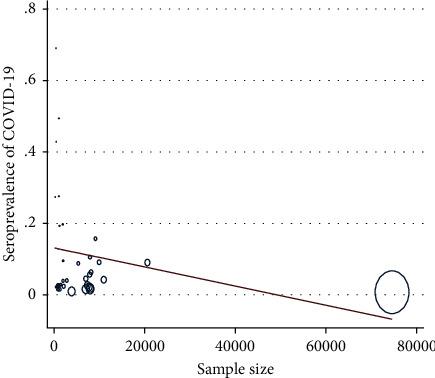
Meta-regression of the relationship between seroprevalence of COVID-19 in blood donors and the sample size.

Meta-regression showed no statistically significant relationships between the seroprevalence of COVID-19 in blood donors and the research sample size (*P* value = 0.213). This does not mean that in studies with larger sample numbers, the seroprevalence of COVID-19 in blood donors is higher (Figure 3).

**Figure 4:**
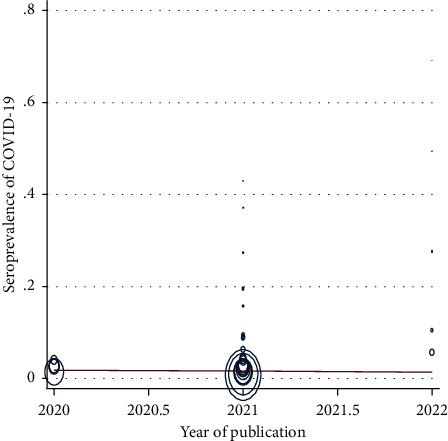
Meta-regression of the relationship between the seroprevalence of COVID-19 in blood donors and the year of publication.

In Figure 4, meta-regression showed no statistically significant relationship between the seroprevalence of COVID-19 in blood donors and the year of publication (*P* value = 0.845). In other words, the seroprevalence of COVID-19 in blood donors has not decreased since 2019.

## 4. Discussion

The seroprevalence of COVID-19 in blood donors was 10%, which is not high. On the other hand, the seroprevalence of IgM and IgG antibodies was high too. About one-quarter of all the blood donors had IgG and about one-third had high IgM. This is worrisome as it indicates that we have had a lot of false-negative tests on blood donors, and this is causing the COVID-19 virus cycle to continue around the world.

In a review study of the European population, the seroprevalence of SARS-CoV-2 in the 12 studies on the general population ranged from 0.42% in Greece to 13.6% in Germany. In 8 blood donor studies, the seroprevalence of SARS-CoV-2 differed from 0.91% in northwestern Germany to 23.3% in Italy [49]. It is essential to carry out a meta-analysis to examine the seroprevalence of SARS-CoV-2 in blood donors given the various results of previous studies.

In a meta-analysis by Kayı et al., the seroprevalence of SARS-CoV-2 was estimated at 8%. The common seroprevalence of the selected variables with higher-than-average rates included male health care workers with 9%, ethnic minority health care workers with 13%, and virus exposure outside of health care (22%) [37]. The seroprevalence of SARS-CoV-2 in the above study is higher than the seroprevalence reported in our meta-analysis. However, in the following article, we see that the seroprevalence of SARS-CoV-2 is lower than the result of the current meta-analysis. Overall, 47 studies, including 392965 cases from 23 countries, were reviewed in a meta-analysis by Rostami et al. to estimate the prevalence of global and regional serology in people with SARS-CoV-2. Its results indicated that the seroprevalence of SARS-CoV-2 in the general population ranges from 0.37 to 1.22%, and this is 3.38% with the collected estimate. At the regional level, the seroprevalence ranged from 1.45% (South America) to 5.27% (Northern Europe) [50]. As is seen, in Rostami's meta-analysis, the prevalence of SARS-CoV-2 in the United States is lower than that in Europe which is in line with the results of our meta-analysis. Race and ethnicity could have a role in the seroprevalence of SARS-CoV-2, but more studies are required to confirm it.

Sharma et al. carried out a study to examine the prevalence of SARS-CoV-2 in Delhi. The adjusted prevalence rate decreased from 28.39% in August to 24.08% in September and reached 24.71% in October [51]. Nonetheless, in our meta-analysis, the seroprevalence of SARS-CoV-2 in blood donors was not statistically declining over time. Vaselli et al. carried out a systematic review with 109 studies involving 17 European countries. Generally, the reported seroprevalence of SARS-CoV-2 was reported to vary from 0.7% to 45.3% among health care workers, with most studies showing no significant differences in terms of gender [52]. However, the prevalence of SARS-CoV-2 was higher in women than men in our meta-analysis.

In Galanis et al. meta-analysis with 49 studies and 127480 subjects, the seroprevalence of SARS-CoV-2 antibodies among healthcare workers was 8.7%. Its prevalence was higher in studies in North America (12.7%) compared to those in Europe (8.5%), Africa (8.2%), and Asia (4%) [53]. Hossain et al. conducted a meta-analysis among 173353 prevaccine health care workers in Europe, the United States, and East Asia. The positive prevalence of IgG antibodies in these regions was 8.6%, in the United States 12.4%, in Europe 7.7%, and in East Asia 4.8% [54]. The seroprevalence of SARS-CoV-2 antibodies in the above two meta-analyses was lower than those of our meta-analysis which could be because of the differences in the study population of these two meta-analyses.

Thirty-three studies involving 10,484 patients were identified in a meta-analysis by Malekifar et al. Simultaneous prevalence of viral infection was 12.58%, blood viruses combined prevalence: 12.48% to 16.93, respiratory viruses combined prevalence: 4.32%. They had up to 6.15 [55]. In a meta-analysis of 23 studies involving 27735 people for determining the prevalence of SARS-CoV-2 antibodies in African countries and related factors, Chisale et al. indicated that the seroprevalence of anti-SARS‐CoV-2 antibodies in Africa was 22% (95% CI: 14–31) [56] that is somehow in line with the results of our meta-analysis reporting a high prevalence.

### 4.1. Study Limitations

1. The age group of the subjects had been reported as age range and the intervals overlapped with each other. Hence, no analyses were carried out based on the age group of the subjects.
2. The lack of uniform distribution of the studies among various countries made the statistics from some countries unavailable.

## 5. Conclusion

The seroprevalence of COVID-19 in blood donors was higher in women than men. Among the blood groups, the highest seroprevalence of COVID-19 was in blood groups O, A, B, and AB, respectively. This shows that the seroprevalence of COVID-19 in blood group O is higher than in other blood groups. From a regional perspective, COVID-19 was most prevalent in Asia, North America, Europe, South America, and Africa. Hence, one can state that Asian race, female gender, and blood type O are the risk factors for the prevalence of COVID-19 in blood donors.

More studies seem to be needed to publish in this regard considering the limitations stated in the studies examined and the limited number of studies published in this regard, so that one can study the seroprevalence of COVID-19 and its antibodies among blood donors with more confidence and in more details.

## Figures and Tables

**Table 1:** Summary characteristics of included articles.

Author	Country	Age group (year)	Sample size	Number of females	Number of males	Prevalence of COVID-19 in total (%)	Prevalence of COVID-19 in females (%)	Prevalence of COVID-19 in males (%)	Date blood donors
Alharbi et al. [24]	Saudi Arabia	26–32	5385	—	—	8.8	—	—	Jun–Nov 2020
Lewin et al. [25]	Canada	>18	7691	3630	4061	2.2	—	—	Between May 25 and July 9, 2020
Valenti et al. [26]	Italy	40.7	789	276	513	2.7	—	—	February 24th to April 8th 2020
Erikstrup et al. [18]	Denmark	17–69	20640	10224	10004	9	—	—	From 6 April to 3 May 2020
Pedersen et al. [27]	Denmark	>70	1201	517	684	1.4	—	—	Between May 16 and May 25, 2020
Pedersen et al. [27]	Denmark	17–69	1110	—	—	2.5	—	—	Between May 16 and May 25, 2020
Stone et al. [28]	USA	>16	9132	4337	4795	15.7	16.8	14.5	March–August 2020
Stone et al. [28]	USA	>16	7986	4057	3929	1.5	1.9	1.1	March–August 2020
Stone et al. [28]	USA	>16	8019	4467	3552	1.9	2	1.7	March–August 2020
Stone et al. [28]	USA	>16	6999	3765	3234	4.5	5.6	3.4	March–August 2020
Stone et al. [28]	USA	>16	11000	5951	5049	4.2	4.2	4.2	March–August 2020
Stone et al. [28]	USA	>16	7000	4046	2954	1.5	9	2.1	March–August 2020
Amorim Filho et al. [29]	Brazil	18–69	2857	1407	1450	4	3.8	4.2	From April 14 to 27, 2020
Cassaniti et al. [30]	Italy		1922	—	—	19.7	—	—	From 18 March to 24 June
Gallian et al. [31]	France	41	998	—	—	2.7	—	—	Last week of March or the first week of April 2020
Pandey et al. [32]	India	25–36	1991	52	1139	9.5	3.8	9.7	From April to July 2020
Mahallawi et al. [33]	Saudi Arabia	18–64	1212	—	—	19.3	—	—	Between mid-May and mid-July 2020
Ng et al. [22]	USA		1000	—	—	0.1	—	—	Mar-20
Sykes et al. [34]	South Africa	15–69	1457	—	—	2.8	—	—	Jan-21
Sykes et al. [34]	South Africa	15–69	463	—	—	2.2	—	—	Jan-21
Sykes et al. [34]	South Africa	15–69	831	—	—	2.4	—	—	Jan-21
Sykes et al. [34]	South Africa	15–69	2107	—	—	2.4	—	—	Jan-21
Slot et al. [35]	Netherlands	18–72	7361	—	—	2.7	2.73	2.7	1–15April 2020
Adetifa et al. [36]	Kenya	15–64	9922	1903	8019	9.1	8.7	9.5	In three periods (30 Apr–19 Jun, 20 Jun–19 Aug, 20 Aug–30 sept)
Runkel et al. [37]	Germany	18–71	3880	1756	2124	0.9	1.1	0.75	Between March and June 2020
Saeed et al. [38]	Canada	>17	74642	35547	39095	0.74	0.72	0.76	Between May 9 and July 21, 2020
Banjar et al. [39]	Saudi Arabia	17–70	837	32	796	1.4	—	1.5	From 20th to 25th May 2020
Sughayer et al. [40]	USA	18–65	292	38	254	27.4	26.3	24	Early February 2021
Jaiswal et al. [41]	India		534			0.429	—	—	Between mid-December 2020 to January 2021
Chaves et al. [42]	Brazil	>16	7837	3553	4284	0.056	—	—	March 1–December 31, 2020
Chunchu et al. [43]	India	18–29	1034	7	1027	0.494	—	—	September 2020 to March 2021
Antonucci et al. [44]	Italy	>18	8183	2047	6136	0.063	—	—	May 2020 to March 2021
Levring et al. [45]	Denmark		105	57	48	0.371	—	—	April 6 to May 28, 2020
Lewin et al. [46]	Canada	51	7924			0.105	—	—	Between January 25, 2021 and March 11, 2021
Kale et al. [47]	India	18–59	1066	18	1048	0.276	—	—	From September to October 2020
Monteon et al. [23]	Mexico	33.5	479			0.691	—	—	August through September 2021
Nesbitt et al. [48]	USA	>15	2008	944	1064	0.039	—	—	From April 27, 2020 – May 11, 2020

**Table 2:** Seroprevalence of COVID-19 in blood donors in the studied subgroups.

Subgroups		Number of study	Prevalence (95% CI)	I^2^ (%)	*P* value
Total		28	10% (9%–11%)	99.6	<0.001

Sex	Male	12	5% (4%–7%)	99.3	<0.001
	Female	12	6% (4%–7%)	99.3	<0.001

Country	Canada	2	4% (1%–8%)	99.8	<0.001
	Germany	1	1% (1%–1%)	0	—
	Denmark	3	9% (4%–15%)	99.4	<0.001
	Saudi Arabia	3	10% (2%–17%)	99.4	<0.001
	USA	3	6% (4%–9%)	99.5	<0.001
	South Africa	1	2% (2%–3%)	0	—
	Italy	3	10% (3%–16%)	99.2	<0.001
	France	1	3% (2%–4%)	0	—
	Netherlands	1	3% (2%–3%)	0	—
	Brazil	2	5% (3%–6%)	92.1	<0.001
	Kenya	1	9% (9%–10%)	0	—
	India	4	32% (12%–52%)	99.6	<0.001
	Mexico	1	69% (65%–73%)	0	—

Continent	North America	7	10% (8%–12%)	99.7	<0.001
	Europe	9	7% (4%–9%)	99.4	<0.001
	Asia	7	23% (14%–31%)	99.6	<0.001
	Africa	2	4% (1%–7%)	98.8	<0.001
	South America	2	5% (3%–6%)	92.1	<0.001

Blood group	A	13	12% (10%–14%)	99.5	<0.001
	B	13	12% (10%–15%)	99.5	<0.001
	AB	13	9% (7%–12%)	99.6	<0.001
	O	13	13% (11%–16%)	99.6	<0.001

Antibody isotypes reported	IgG	9	23% (18%–29%)	99.8	<0.001
	IgM	5	29% (9%–49%)	99.8	<0.001

## Supplementary Materials

Supplementary Materials Supplementary MaterialsTable s1: search strategy in electronic databases. Table s2: Newcastle Ottawa Scale checklist.
