# Supplementary material for: Seroprevalence of COVID-19 in Blood Donors: A Systematic Review and Meta-Analysis
Source: Adv Virol. 2022 Jul 21;2022:9342680. doi: 10.1155/2022/9342680 (PMC9334089; doi:10.1155/2022/9342680)
Supplement: Supplementary Materials — Table s1: search strategy in electronic databases. Table s2: Newcastle Ottawa Scale checklist. [file 9342680.f1.docx]

**Appendix**

Table s1: Search strategy in Electronic databases.

| Electronic databases | Search strategy |
| --- | --- |
| PubMed | ((SARS-Cov-2 OR COVID-19 OR Coronavirus-2) AND (Seroprevalence)) AND (Blood donors) |
| Scopus | ( TITLE-ABS-KEY ( blood  AND donors )  AND  TITLE-ABS-KEY ( sars-cov-2  OR  covid-19  OR  coronavirus-2 )  AND  TITLE-ABS-KEY ( seroprevalence ) ) |
| Web of Science | (COVID-19 OR "coronavirus disease 2019" OR SARS-CoV-2) AND TOPIC: (Blood donors) AND TOPIC: (Seroprevalence)  Timespan: 2019-2021. |

Table s2: Newcastle Ottawa Scale checklist.

| Studies | Q1 | Q2 | Q3 | Q4 | Q5 | Q6 | Q7 | Q8 |
| --- | --- | --- | --- | --- | --- | --- | --- | --- |
| Alharbi NK, 2021 | * | * | * | * | * | * | * |  |
| Lewin A, 2021 | * | * | * | * |  | * | * | * |
| Valenti L, 2021 | * | * |  |  | * | * | * | * |
| Erikstrup C, 2021 |  | * | * | * | * | * | * |  |
| Pedersen OB, 2020 | * | * |  | * | * |  | * | * |
| Stone M, 2021 |  | * | * | * |  | * | * | * |
| Amorim Filho L, 2020 | * | * | * |  | * |  | * | * |
| Cassaniti I, 2021 |  | * | * | * | * | * | * | * |
| Gallian P, 2020 | * | * | * | * | * | * | * | * |
| Pandey HC, 2021 | * | * | * | * | * | * | * |  |
| Mahallawi WH, 2021 |  | * | * | * | * | * | * | * |
| Ng DL, 2020 | * | * | * | * | * | * | * | * |
| Sykes W, 2021 | * | * |  |  | * | * | * | * |
| Slot E, 2020 | * | * | * | * |  |  | * | * |
| Adetifa IM, 2021 |  | * | * | * | * | * | * | * |
| Runkel S, 2021 | * | * | * | * | * | * | * |  |
| Saeed S, 2021 | * | * | * |  | * | * | * | * |
| Banjar A, 2021 | * | * |  | * | * | * | * | * |
| Sughayer MA, 2021 | * | * | * | * | * |  | * | * |
| Jaiswal R 2021 | * | * | * |  | * |  | * | * |
| Chaves DG 2022 |  | * | * | * | * | * | * | * |
| Chunchu, S. R 2022 | * | * | * | * |  |  | * | * |
| Antonucci F, 2021 |  | * | * | * | * | * | * | * |
| Levring MB, 2021 | * | * | * | * | * | * | * |  |
| Lewin A, 2022 | * | * | * |  | * | * | * | * |
| Kale P, 2022 | * | * |  | * | * | * | * | * |
| Nesbitt DJ, 2021 | * | * | * | * | * |  | * | * |
